# Differential lung ventilation for increased oxygenation during one lung ventilation for video assisted lung surgery

**DOI:** 10.1186/s13019-019-0910-2

**Published:** 2019-05-06

**Authors:** Ran Kremer, Wisam Aboud, Ori Haberfeld, Maruan Armali, Michal Barak

**Affiliations:** 1 Department of Thoracic Surgery, Rambam Health Care Campus, Haifa, Israel; 2 Department of Anesthesiology, the Baruch Padeh Medical Center, Poriya, Tiberius, Israel; 3 Department of Anesthesiology, Rambam Health Care Campus and the Rappaport Faculty of Medicine, Technion - Israel Institute of Technology, POB 9602, 31096 Haifa, Israel

**Keywords:** One lung ventilation, Continuous positive airway pressure, Differential lung ventilation

## Abstract

**Background:**

One lung ventilation (OLV) is the technique used during lung resection surgery in order to facilitate optimal surgical conditions. OLV may result in hypoxemia due to the shunt created. Several techniques are used to overcome the hypoxemia, one of which is continuous positive airway pressure (CPAP) to the non-dependent lung. Another technique is ventilating the non-dependent lung with a minimal volume, thus creating differential lung ventilation (DLV). In this study we compared the efficacy of CPAP to DLV during video assisted thoracoscopic lung resection.

**Patients and method:**

This is a prospective study of 30 adult patients undergoing elective video assisted thoracoscopic lung lobectomy. Each patient was ventilated in four modes: two lung ventilation, OLV, OLV + CPAP and OLV + DLV. Fifteen patients were ventilated with CPAP first and DLV next, and the other 15 were ventilated with DLV first and then CPAP. Five minutes separated each mode, during which the non-dependent lung was open to room air. We measured the patient’s arterial blood gas during each mode of ventilation. The surgeons, who were blinded to the ventilation technique, were asked to assess the surgical conditions at each stage.

**Results:**

Oxygenation during OLV+ CPAP was significantly lower that OLV + DLV (*p* = 0.018). There were insignificant alterations of pH, PCO2 and HCO3 during the different ventilating modes. The surgeons’ assessments of interference in the field exposure between OLV + CPAP or OLV + DLV was found to be insignificant (*p* = 0.073).

**Conclusions:**

During OLV, DLV is superior to CPAP in improving patient’s oxygenation, and may be used where CPAP failed.

**Trial registration:**

ClinicalTrials.gov NCT03563612. Registered 9 June 2018, retrospectively (due to clerical error).

## Introduction

During lung resection surgery, optimal surgical access is attained when the operated lung is deflated and its movements are avoided. This is achieved by one lung ventilation (OLV) [1]. Ventilation of one lung creates a trans-pulmonary shunt through the non-ventilated lung and causes hypoxemia [2, 3]. Both mechanical factors, such as gravitation and pressure by the surgeon, and the physiological response, mainly hypoxic pulmonary vasoconstriction, decrease the shunt [4, 5]. The hypoxemia is usually not severe; however, in some cases, life threatening hypoxemia occurs that responds poorly to corrective maneuvers [6]. Applying positive end expiratory pressure (PEEP) to the dependent lung and ventilating with 100% oxygen, are initial steps. In case there is no improvement in oxygenation, additional techniques are used. One technique is insufflating oxygen with a constant pressure to the non-dependent lung, called continuous positive airway pressure (CPAP). Another option is to ventilate the non-dependent lung with a minimal volume and rate, creating differential lung ventilation (DLV). Both techniques may impair exposure to the operated area to some extent.

The purpose of this study is to compare ventilation modalities during OLV, which may improve oxygenation with minimal impairment of the surgical field conditions. In this study, the non-dependent lung was ventilated alternately, in crossover fashion, by CPAP and by a portable ventilator with low rate and pressure in a DLV technique [7–9]. We anticipated that a low ventilation rate and pressure would produce the least interference with the surgeon’s exposure and, at the same time, improve oxygenation.

## Patients and methods

This is a prospective randomized controlled crossover study of adult patients scheduled to have video-assisted thoracoscopic surgery (VATS) of lung lobectomy under general anesthesia. The study was approved by the institution local Ethic Committee and registered (ClinicalTrials.gov NCT03563612). The patients had a detailed explanation of the study before the surgery by one of the anesthesiologists who took part in this study, and signed an inform consent if they agreed to participate.

Exclusion criteria included: American Society of Anesthesiologists (ASA) grade ≥ 4, pregnancy, and difficult intubation. Decreased oxygen saturation below 85% at any time during the surgery was set as an end point, at which the study would be stopped. The primary outcome variables are the PaO_2_ measurements, while the secondary outcome variables are the spirometry measurements and the surgeons’ evaluation of the surgical field.

### Study protocol

On arrival in the operating room, an intravenous line was placed. Each patient was monitored with an electrocardiogram, pulse oximeter, invasive blood pressure, end tidal capnography, and eosophageal thermometer. Following the induction of general anesthesia using fentanyl 2–5 microgram/kg; propofol 1–3 mg/kg and rocuronium 0.6–0.8 mg/kg, the trachea was intubated with a left double lumen tracheal tube VivaSight (ETView Ltd. Misgav Business Park, Israel) where verification of the tube position was monitored continuously with on-line video surveillance. The dependent lung was ventilated by anesthesia machine (Dräger Narkomed 2A) with sevoflurane for maintenance of anesthesia.

Ventilation parameters were as follows: volume controlled mode with tidal volume was set to 8 ml/kg during two lung ventilation (TLV) and reduced to 6 ml/kg during OLV; respiratory rate of 10–12 breaths per minute during TLV, increased to 12–15 per minute during OLV, adjusted to keep P_a_CO_2_ below 50 mmHg; inspired oxygen of 100% at all times; positive end expiratory pressure (PEEP) was 2 cm H_2_O, first in both lungs and then in the dependent lung during OLV. All patients were placed in the lateral decubitus position for surgery.

When OLV was initiated, the non- dependent lung was open to the atmosphere. Ten minutes after the first trocar was introduced, measurements were recorded and then the operated lung was either connected to a CPAP system or to a small portable time-cycled ventilator paraPAC-2D (Transport Ventilator, SIMS pneuPAC Ltd., Luton, UK). The order of intervention was randomized by one of the researchers (MB), using computerized software (random.org). The CPAP pressure was set to 5 cm H_2_O. Differential Lung Ventilation of the non-dependent lung was set at a rate of 8 breaths per minute, inspired gas 100% oxygen, peak pressure and tidal volume set to the lowest available values, resulting in a peak pressure of 10 cm H_2_O and a tidal volume around 50 ml. The treatment of the operated lung was alternated with a 5-min interval between modes of ventilation, without additional ventilatory support or oxygen insufflations, to avoid the influence of one modality upon the other. In that 5 min interval, the tube connection of the non-dependent lung was opened to room air.

### Measurements

Demographic data regarding the patients’ age, weight, gender and ASA classification was recorded. Arterial blood gas, peak inspiratory pressure (PIP), and plateau pressure (Pplat) were measured during: two lung ventilation, OLV (10 min after the first trocar was introduced), OLV+ CPAP and OLV+ DLV. PIP and Pplat were recorded from the anesthesia machine spirometer. At the same time, the chief surgeon was asked to comment on the surgical field conditions. The surgeon’s evaluation was graded from 0 (no interference) to 3 (maximal interference). The surgeons were blinded to the ventilation mode used at that time since the patient’s sterile covers were pulled up, concealing the anesthesia machine and the ventilator.

### Statistical analysis

Sample size was calculated assuming difference in P_a_O_2_ between CPAP and mini-ventilation of 50 mmHg; a safety level of 95%; standard deviation of 60; intensity of 80% and measurement ratio of 1:1. Comparison between the groups of patients was performed using the Mann Whitney non-parametric test. Data of the arterial blood gas and spirometry variables during the different ventilation techniques were compared with Wilcoxon non-parametric test. Surgeons’ estimations of interference with the surgical field were compared with the chi-square test. Differences were considered statistically significant at *p* < 0.05.

## Results

Thirty patients were recruited as participants. One patient in the group that had DLV first was excluded, due to difficulty in tracheal intubation. No significant difference was found between the two groups, CPAP first or DLV first, in all studied variables. Participant’s demographics are shown in Table 1 and data regarding the surgery in Table 2.

**Table 1:** Patients’ demographics

Age, years	65 ± 10^a^
Male/Female	18/11
Weight, kg	82 ± 19^a^
BMI	28 ± 5^a^
ASA 2/3/4	13/15/1

Note

^a^Data presented in mean ± standard deviation

**Table 2:** Data regarding the operation

	CPAP first (*n* = 15)	DLV first (*n* = 14)	*P* value
Lobe resected:			
RUL/RLL/LUL/LLL	5/4/5/1	4/4/3/3	0.53
Length of surgery (min)	130 ± 34 *	137 ± 48 *	0.74

Note

* Data presented in mean ± standard deviation

**Fig. 1:**
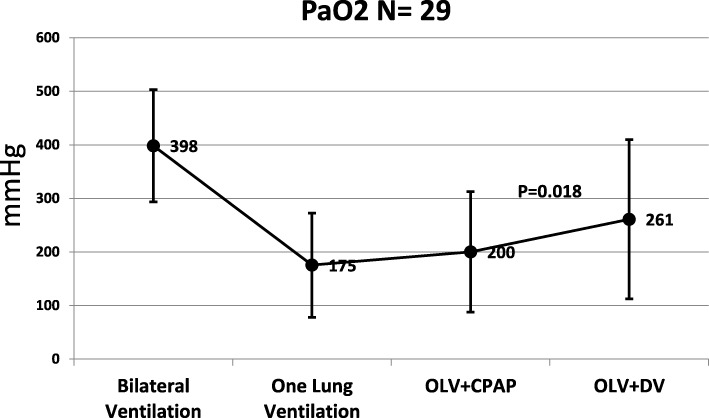
Arterial PaO_2_

**Fig. 2:**
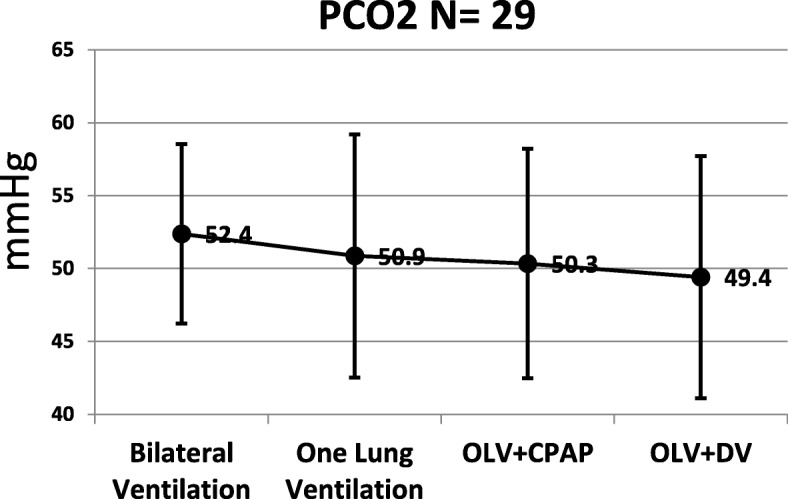
Arterial PaCO_2_

**Fig. 3:**
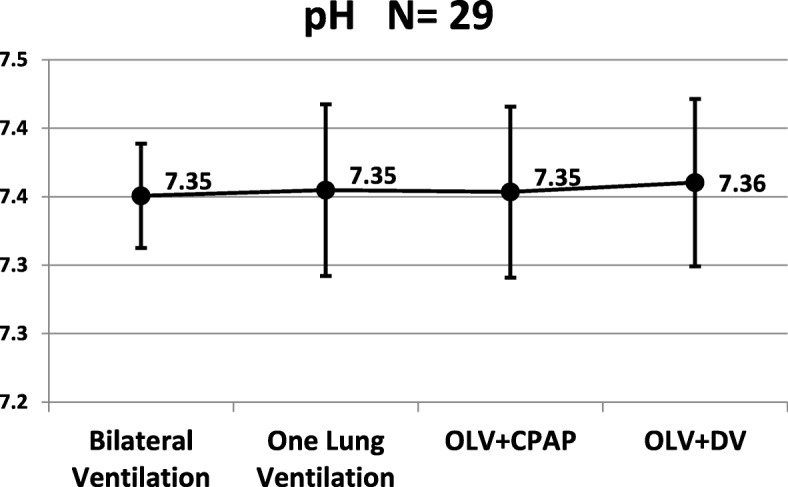
Arterial pH

**Fig. 4:**
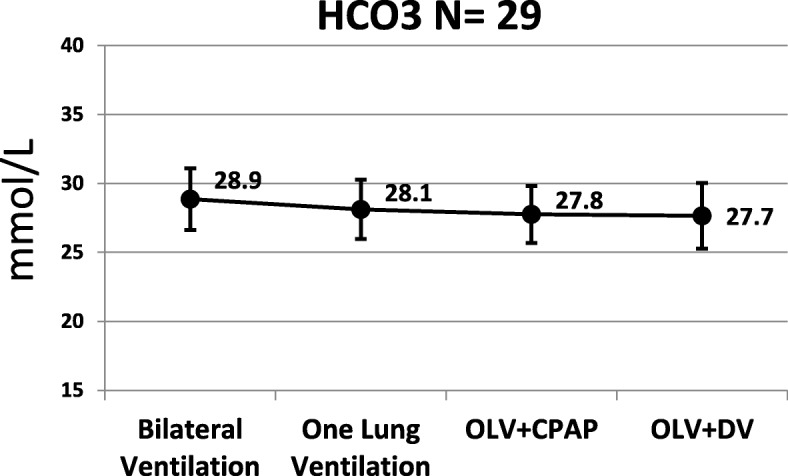
Arterial HCO_3_. B = two lung ventilation. A = one lung ventilation. C = one lung ventilation + CPAP. D = one lung ventilation + DLV

Regarding arterial blood gas: oxygenation reduced significantly when changing from two lung ventilation to OLV (Fig. 1). Oxygenation during OLV+ CPAP was significantly lower that OLV + DLV (*p* = 0.018). Alterations of PCO_2_, pH and HCO_3_ during the different ventilating modes were not significant (Figs. 2, 3, 4 respectively).

No significant difference was noted between peak and plateau pressures during OLV, OLV + CPAP or OLV + DLV.

Regarding the surgeons’ assessment of interference in surgical field exposure:

None of the patients was graded as 3, where interference is maximal. One patient was graded as 1 during CPAP and 2 during DLV. One patient was graded as 2 for both techniques. The rest of the patients received 0 interference for CPAP while six had 1 grade interference with DLV (*p* = 0.073).

## Discussion

In this study we found that DLV is superior to CPAP as mean for improving patient’s oxygenation during OLV. The theoretical explanation for DLV superiority relies on the physiologic phenomenon of heterogeneity in different areas in the lung [10, 11]. Ventilating both lungs with the same pressure results in fresh gas flow ventilating the lower resistance parts of the lung, while the areas with high resistance, such as atalectatic areas, remain unventilated. Heterogeneous lung aeration may result in lung inflammation and injury, which deteriorates gas exchange furthermore [12, 13]. Ventilating independently different parts of the lung with different pressures may help force the air into atalectatic parts, reduce V/Q mismatch and improve oxygenation [14, 15]. This theory is supported by experimental models [7, 12]. Clinical trials showed a similar beneficial effect of DLV in patients in intensive care units [16–18] and during open thoracic surgery [9, 19]. However, we found no data regarding DLV during thoracoscopic surgery.

Theoretically, changing ventilation may improve oxygenation indirectly by reducing CO_2_ levels, according to the formula: PaO_2_ = FiO_2_ (Pbr-PH_2_O)-PCO_2_/K. However, we found no significant change in CO_2_ in this study. Thus, improved oxygenation was not the outcome of CO_2_ levels.

The main disadvantage of DLV, as with CPAP, is that both may interfere with surgical field exposure. Spirometry of the dependent lung was recorded in order to find whether the different ventilation modes of the non-dependent lung influence the dependent one. No significant difference was found.

An important weakness of this study relies on its design as a cross-over study. There is a possibility of carry-over effects and its influence on interpretation of the findings. In order to overcome it we set a period of 5-min interval between modes of ventilation, without additional ventilatory support or oxygen insufflations. In that 5 min interval, the tube connection of the non-dependent lung was opened to room air. We assume that this time interval was long enough to allow patient’s oxygenation return to its baseline. Another problem was the non-significant results of the surgical exposure assessment (*p* = 0.073). This marginally non-significant result may be caused by Type II error, and may have been different in a larger scale study. The surgeons were blinded to the mode of ventilation, yet the anesthesiologist was not. This may have set additional error to the study.

In the past decades, when cardio-thoracic surgery became minimally invasive, good lung deflation became a necessity. Video- and robot- assisted thoracoscopic operations require high quality OLV [20, 21]. Moreover, many of these procedures are performed with the patient in the supine position, with a large shunt and low oxygenation. At the same time, the patient often has poor basic lung functions, and hypoxemia occurs rapidly. Patients with ischemic heart disease who undergo coronary artery bypass graft are especially susceptible to injury during hypoxemia, and aggressive treatment of arterial desaturation is mandatory to ensure the patient’s safety [22, 23]. The challenge of the anesthesiologist is to overcome hypoxemia without disturbing exposure of the surgical field. We believe the DLV is an additional tool for improving oxygenation during OLV.

## Conclusions

The use of DLV while ventilating one lung may improve patient’s oxygenation, and was found to be better than CPAP. Differential lung ventilation may be used where CPAP failed.

## Abbreviations

ASA: American Society of Anesthesiologists
CPAP: Continuous positive airway pressure
DLV: Differential lung ventilation
OLV: One lung ventilation
PEEP: Positive end expiratory pressure
PIP: Peak inspiratory pressure
Pplat: Plateau pressure
VATS: Video-assisted thoracoscopic surgery
